# Continuous Cardiac Troponin I Release in Fabry Disease

**DOI:** 10.1371/journal.pone.0091757

**Published:** 2014-03-13

**Authors:** Andreas Feustel, Andreas Hahn, Christian Schneider, Nicole Sieweke, Wolfgang Franzen, Dursun Gündüz, Arndt Rolfs, Christian Tanislav

**Affiliations:** 1 Department of Internal Medicine, Justus Liebig University, Giessen, Germany; 2 Department of Child Neurology, Justus Liebig University, Giessen, Germany; 3 Department of Radiology, Justus Liebig University, Giessen, Germany; 4 Department of Neurology, Justus Liebig University, Giessen, Germany; 5 Department of Cardiology, Justus Liebig University, Giessen, Germany; 6 Albrecht-Kossel Institute for Neuroregeneration, University of Rostock, Rostock, Germany; Julius-Maximilians-Universität Würzburg, Germany

## Abstract

**Background:**

Fabry disease (FD) is a rare lysosomal storage disorder also affecting the heart. The aims of this study were to determine the frequency of cardiac troponin I (cTNI) elevation, a sensitive parameter reflecting myocardial damage, in a smaller cohort of FD-patients, and to analyze whether persistent cTNI can be a suitable biomarker to assess cardiac dysfunction in FD.

**Methods:**

cTNI values were determined at least twice per year in 14 FD-patients (6 males and 8 females) regularly followed-up in our centre. The data were related to other parameters of heart function including cardiac magnetic resonance imaging (cMRI).

**Results:**

Three patients (21%) without specific vascular risk factors other than FD had persistent cTNI-elevations (range 0.05–0.71 ng/ml, normal: <0.01). cMRI disclosed late gadolinium enhancement (LGE) in all three individuals with cTNI values ≥0.01, while none of the 11 patients with cTNI <0.01 showed a pathological enhancement (p<0.01). Two subjects with increased cTNI-values underwent coronary angiography, excluding relevant stenoses. A myocardial biopsy performed in one during this procedure demonstrated substantial accumulation of globotriaosylceramide (Gb_3_) in cardiomyocytes.

**Conclusion:**

Continuous cTNI elevation seems to occur in a substantial proportion of patients with FD. The high accordance with LGE, reflecting cardiac dysfunction, suggests that cTNI-elevation can be a useful laboratory parameter for assessing myocardial damage in FD.

## Introduction

Fabry disease (FD) is a rare X-linked disorder affecting hemizygous males and heterozygous females. Deficiency of the lysosomal enzyme alpha-galactosidase A (α-GAL-A) results in accumulation of glycosphingolipids with terminal alpha-galactosyl residues in various organs and tissues, in particular in the central and peripheral nervous system, vessels and kidneys. Approximately 60% of patients with FD have cardiac involvement such as ventricular hypertrophy, valvular heart disease, conduction defects, or coronary artery disease [1], [2]. These alterations predispose to congestive heart failure, arrhythmia, and myocardial infarction, and can determine the prognosis of the disease [3]–[5]. In FD, assessment of disease burden by specific biomarkers is crucial, since this can disclose preclinical organ involvement, and can help to make therapeutic decisions timely [6]–[8].

Cardiac troponin I (cTNI) is a laboratory parameter well known to reflect acute and chronic cardiac muscular damage [9]. Recently, we noticed a continuously elevated cTNI in a FD patient with cardiac involvement, but without coronary artery disease [10]. The purpose of this study was to investigate whether cTNI can be a suitable biomarker for assessment of the cardiac status in FD. To accomplish this, we determined cTNI levels in a smaller cohort of patients with FD and related the data to other parameters of cardiac function.

## Patients and Methods

### Patients

The study cohort included 14 patients with FD treated and followed-up in our centre (6 males and 8 females). Specific parameters such as enzyme activity (α-GAL-A reference range 33.2–109 nmol MU/h/mg protein) and levels of Lyso-globotriaosylceramide in serum (Lyso-Gb_3_, values >0.5 ng/ml were interpreted as increased) were determined in all patients. The definite diagnosis of FD had been confirmed by molecular genetic analysis demonstrating a heterozygous or hemizygous mutation in the α-GAL-A-gene [11]. Specific signs and symptoms as characteristic for FD such as angioceratoma, cornea verticillata, gastrointestinal symptoms, and any other clinical signs related to FD pathology were recorded systematically.

Age at last follow-up ranged from 21–80 years (median 50 years). 7 subjects received enzyme replacement therapy with recombinant alpha galactosidase A. In all patients the routine follow-up protocol encompassed laboratory testing including cTNI determination at least twice per year, assessment of the patient’s overall condition and his neurological, cardiac, and nephrological status. Magnetic Resonance Imaging (MRI) of the brain and the heart was done annually.

### Ethics Statement

The study protocol was reviewed by the ethical committee of the medical faculty of the Justus Liebig University Giessen. To use routine clinical data the ethical board recommend obtaining patient’s written informed consent from each participant if possible. In 13 patients a written consent was obtained, one patient could not be contacted any longer. The ethical board approved the conduction of the analysis.

### Cardiac Assessment

cTNI, was measured by the ADVIA Centaur® TnI-Ultra immunoassay (Bayer HealthCare, Tarrytown USA). A cTNI cut-off level of ≥0.01 ng/ml was considered as relevant elevation. Furthermore, brain natriuretic peptide (BNP) was determined in all patients. Values >37 pg/ml were considered as abnormal.

In all patients, a routine electrocardiogram (ECG), a Holter-ECG and a cardiac ultrasound were performed. Arrhythmia was diagnosed if one of the following symptoms was detected in Holter-ECG: persistent or intermittent atrial fibrillation or flatter, sustained tachycardia (heart rate ≥100/minute for more than 30 seconds), non-sustained tachycardia (heart rate ≥100/minute for less than 30 seconds in at least 3 subsequent heart cycles), incomplete bundle branch block (QRS-duration: 100–119 ms) or complete bundle branch block (QRS-duration ≥120 ms).

Cardiac MRI findings were analyzed by an experienced radiologist blinded from any clinical data. The following parameters were systematically assessed: left ventricular ejection fraction (LVEF), left ventricular (LV) hypertrophy, right ventricular (RV) hypertrophy, left ventricular posterior wall thickness measured during diastole (LVPWd), interventricular septum thickness during diastole (IVSd), LV mass index, and late gadolinium enhancement (LGE). The LGE technique (8-mm slice thickness, breath hold, short heart axis) was applied to detect changes in tissue integrity in the left ventricle. Short-axis views at the basal, mid, and apical segments were used for the semiquantitative assessment of the appearance of gadolinium enhancement in every left ventricular segment indicating the occurrence of intramyocardial fibrosis.

### Nephrological Assessment

Glomerular filtration rate (eGFR) was calculated according to the simplified MDRD equation: eGFR (ml/min/1,73 m2 body surface) = 186×(serum creatinine)−1.154×(age) −0.203×0.742 (if female)×1.212 (if black) [12]. In addition, protein and albumin excretion was determined in a 24 h urine sample. The reference range for proteinuria was <159 mg/24 h, and that for albuminuria was <30 mg/24 h.

### Neurological Assessment

A thorough neurological examination was performed in all patients at least twice per year. Small fibre dysfunction was assessed by quantitative sensory testing. Affection status of the brain supplying arteries was determined by measuring intima-media thickness in the common carotid artery by means of transcranial Doppler sonography. In addition, all patients underwent a brain MRI for assessment of white matter lesions.

### Statistical Analysis

Data are presented as median values and range. cTNI levels were related to the nephrological data and various cardiac and cardiac MRI parameters obtained at last follow-up examination. Cardiac MRI and other parameters were compared between patients with cTNI values ≥0.01 ng/ml and those with normal values (<0.01 ng/ml).

## Results

**Figure 1:**
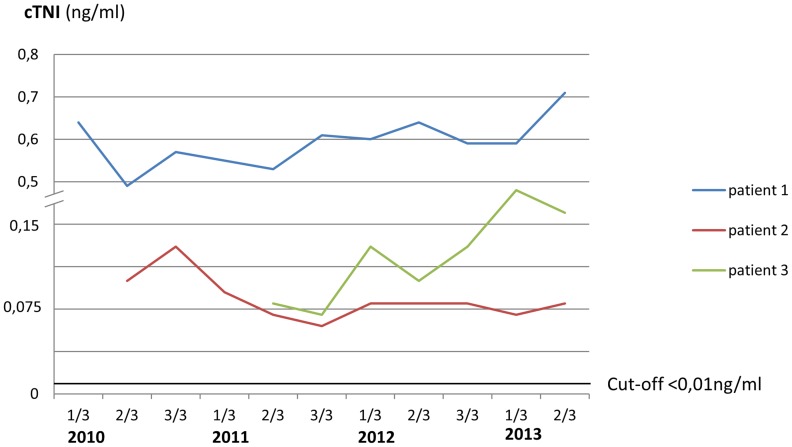
cTNI-values in 3 patients with Fabry disease.

**Figure 2:**
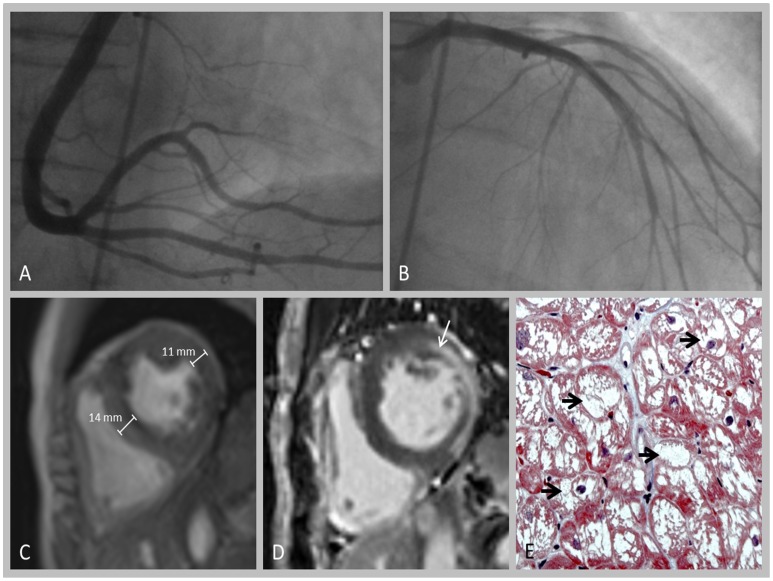
Cardiac work up in patient 2. A+B: Coronary angiography (A: right coronary artery; B: left coronary artery) demonstrating no relevant pathology. C+D: Cardiac MRI showing increase in myocardial wall thickness (C) and pathological late gadolinium enhancement (D, arrow). E: Myocardial biopsy revealing strong accumulation of Gb_3,_ as indicated by numerous vacuoles within the cardiomyocytes (arrow).

The underlying mutations in all 14 patients are summarized in table 1. In 3 out of 14 patients (2 females and one male) (21%), repetitive cTNI assessments (3–5 times per year) demonstrated continuously elevated values, reflecting persistent cTNI-elevation (Figure 1). Anthropometrical and clinical data, medical history, and laboratory and cardiac findings of these three patients in relation to subjects without cTNI-elevation and to normal values are summarized in table 2. All three individuals with elevated cTNI levels were treated by ERT since 5, 3, and 6 years, respectively; while cTNI-measurements were started 5, 3 and 4 years after initiation of ERT. cTNI in subjects with increased values ranged from 0.06 to 0.71 ng/ml. None of them had vascular risk factors other than FD, including hypertension, diabetes and hyper-cholesterolemia. One patient temporarily complained of symptoms compatible with angina pectoris, whereas none of them had an acute coronary syndrome or stroke prior to or after making the diagnosis of FD. A coronary angiography performed in 2 of them did not reveal coronary artery stenoses. Patient 2 underwent a myocardial biopsy, demonstrating a relevant accumulation of Gb_3_ in cardiomyocytes (Figure 2).

**Table 1:** Synopsis of alpha-galactosidase gene mutations in 14 patients included in the study.

Mutation	Patients
c.424>C [C142R]	Patient 1
c.514T>G [C172G]	Patient 2
c.994dupA [p.Arg332Lysfs*7]	Patient 3
c.755G>C [R252T]	1 female, 2 males
c.376A>G [S126G]	2 females, 2 males
c.1025G>A [R342Q]	1 female, 1 male
c.416A>G [N139S]	1 female
c.376A>G [s126G]	1 female

**Table 2:** Baseline data, medical history, biomarkers and cardiac work up in patients with FD in relation to cardiac troponin I elevation and normal values.

	Patient 1	Patient 2	Patient 3	FD-patients without cTNIelevation (n = 11)
**cTNI** (ng/ml); median (range)	0.59 (0.49–0.71)	0.08 (0.06–0.13)	0.073 (0.07–0.18)	<0.01
**Baseline data**				
Age	61	59	35	50 (21–80)
Male	No	No	Yes	5
BMI (kg/m^2^)	19.8	19.3	22.4	23.6 (20.4–30.8)
**Medical history**				
Hypertension	No	No	No	0
Diabetes mellitus	No	No	No	0
Hypercholesterolemia (n = 9)	No	No	No	0
Current Smoker	No	No	No	0
Peripheral artery occlusive disease	No	No	No	0
Angina pectoris	Yes	No	No	0
Intima media thickness	No	No	No	0
Acute coronary syndrome	No	No	No	0
Previous stroke	No	No	No	3
Angioceratoma	No	Yes	Yes	3
Cornea verticillata	Yes	Yes	Yes	1
Gastrointestinal symptoms	Yes	Yes	Yes	2
Small fibre neuropathy*	Yes	Yes	Yes	3
White matter lesions (cerebral MRI)	Yes	n.d.	No	5
**Clinical chemistry**				
α-GAL-A activity (nmol MU/mg protein); (reference range >33); (n = 13)	5	2.6	0.02	49 (26–63)
Serum-Lyso-Gb3 (ng/ml) (reference range <0.5), (n = 13)	15.2	21.7	67.1	0.1 (LLQ§-0.73)
Brain natriuretic peptide (pg/ml); (reference range <37); (n = 12)	161	288	15	16 (2–90)
Serum-Creatinin (mg/ml)	1.1	0.7	1.2	0.8 (0.7–1)
eGFR(ml/min/1.73 m^2^)	53.4	88.7	73.2	84.4 (64.9–151.3)
Proteinuria (mg/24 h); (reference range <159 mg/24 h)	1141	1062	318	81.8 (52–160.2)
Albuminuria (mg//24 h); (reference range <30 mg/24 h)	657	739	204	21 (17–30.9)
**Cardiac MRI (n = 13)**				
LVEF (%); (reference range ≤60%)	77	65	64	62 (46–70)
LV-hypertrophy/concentric	Yes	Yes	Yes	0
LV-hypertrophy/eccentric	No	No	No	0
RV-hypertrophy	No	No	No	0
Ventricular wall thickness (mm); (reference range ≤11 mm)†	14	11	10	8 (6–11)
Ventricular Septum (mm); (reference range ≤11 mm)	14	14	8	7 (5–10)
LV-mass index (g/m^2^) (reference range ≤115 g/m^2^ inmales and ≤95 g/m^2^ in females)	133.4	92	141.9	101 (65.2–139.5)
LGE	1	1	1	0
**Electrocardiogram**				
Sinus rhythm	Yes	Yes	Yes	11
PQ-duration (ms); (reference range ≤200 ms)	216	116	134	138 (116–164)
QRS-duration (ms); (reference range ≤100 ms)	90	110	94	88 (76–112)
QT-duration (ms); (reference range <460 ms)	411	415	384	397 (370–432)
**Holter**				
Arrhythmia$	1	1	0	1
**Pacemaker**	No	No	No	1

*a small fibre dysfunction was proved by quantitative sensory testing or by skin biopsy.

† measurement end-diastolic in the posterior wall of the left ventricle.

$ Arrhythmia was considered if one of the following conditions was detected: persistent or intermittent atrial fibrillation of flatter, sustained tachycardia (heart rate ≥100/minute for more than 30 seconds), non-sustained tachycardia (heart rate ≥100/minute for less than 30 seconds in at least 3 subsequent hear cycles), incomplete bundle branch block (QRS-duration: 100–119 ms) or complete bundle branch block (QRS-duration ≥120 ms).

§ lower level of quantification.

All FD patients revealed values for the LVEF within normal ranges. One patient showed left ventricular hypertrophy and 5 had elevated BNP-values. Ventricular tachycardias were detected by Holter-ECG in 3 patients, necessitating pacemaker implantation in one. Comparison of cardiac MRI parameters between patients with and without cTNI elevation revealed higher LVPWd thickness in those with increased cTNI levels, whereas IVSd and EF did not differ between the two groups. Importantly, all three patients with elevated cTNI values showed LGE, whereas this was not the case in any of the other subjects. The three patients with increased cTNI-levels had concentric left ventricular hypertrophy, two showed distinctly increased IVSd thickness (both 14 mm). All three subjects with cTNI elevation had proteinuria and albuminuria, while eGFRs were not relevantly reduced (53.4–88.7 ml/min/1.73 m2).

During follow-up, in the patients with continuously increased cTNI-values, serial echocardiographic examinations revealed no significant changes of cardiac wall thickness (data not shown).

## Discussion

The main findings of this study were that approximately 20% of patients in this FD cohort had continuously increased cTNI values, and that cTNI elevation was highly correlated with pathologic LGE in the cardiac MRI.

In FD it is assumed that the cardiac pathology is mainly related to progressive cardiac hypertrophy [13]. Storage of Gb_3_ has been demonstrated in various structures of the heart including cardiomyocytes, cells of the conduction system, fibroblasts and endothelium cells within all types of vessels [14]. The proposed pathophysiological mechanism is an absolute and relative ischemia determined by cardiac hypertrophy on one hand, and angiopathy of small arteriols and capillaries on the other [14], [15].

In two of our patients with elevated cTNI values, coronary macroangiopathy could be ruled out by angiography, performed to clarify cTNI elevation. Persistent cTNI elevation secondary to small vessel stenosis also seems unlikely, because no clinical signs and no electrocardiographic abnormalities characteristic of myocardial ischemia were detected. Furthermore, distribution of LGE within the myocardial wall does not support the hypothesis of an ischemic mechanism in these subjects. Therefore, a direct damage of cardiomyocytes by abnormal Gb_3_ storage appears to be the most likely cause of persistent cTNI elevation. This assumption is also supported by the marked accumulation of Gb_3_ in cardiomyocytes as observed in the myocardial biopsy of patient two (Figure 2).

Since cTNI elevation is usually considered highly specific for myocardial damage, this finding often prompts further examinations such as coronary angiography. Therefore, the observation of increased cTNI-values in FD patients without coronary artery disease is of clinical relevance, since it may help to avoid nonessential invasive procedures [9], [16].

However, when interpreting our results, potential causes for cTNI elevation other than cardiac pathology needs to be taken into account. Elevated cTNI values not related to myocardial damage have been described in patients with critical illness, sepsis, stroke, and seizures [9], [17]–[24]. The latter two causes are typical central nervous system symptoms complicating FD. But since none of our three patients with continuously increased cTNI levels suffered from stroke or epilepsy, these mechanisms can be excluded. Measuring cTNI by immunoassays as done in our study has been identified as a potential source of false positive results [22]. However, as cTNI determinations were performed in all our patients with the same test and device, and since only subjects with LGE in cardiac MRI had elevated values, this makes a systematic analytical error in our cohort unlikely.

Further, it has been shown that renal insufficiency can result in elevated cTNI values [22], [25]–[29]. Indeed, all three subjects with cTNI-elevation in our study group also showed signs of renal involvement such as reduced GFR, proteinuria, albuminuria, or mildly increased creatinine values. Brunet and colleagues investigated cTNI alterations in patients with end-stage renal disease [25]. Comparing their data with those obtained in our subjects with relatively well preserved renal function, shows similar cTNI levels in two of our individuals, while the third one had even distinctly higher values than patients with end-stage renal disease. But collectively, it can not be excluded definitely that renal impairment contributed additionally to continuous cTNI-elevations in our patients. Conversely, our findings highlight that FD is a differential diagnosis in patients with otherwise unexplained cTNI elevations.

Late gadolinium enhancement in cMRI is a valid and sensitive method for visualizing myocardial interstitial abnormalities in FD [30], [31]. In this study all subjects with cTNI elevation also had LGE, whereas this was not the case in any of the other patients, thus pointing to considerable convergence between cTNI-values and cardiac pathology in FD.

Recently, a correlation between genotype, phenotype and clinical severity in FD has been emphasized [32]. Lyso-Gb_3_, is a biomarker considered to be closely related to disease severity [32]. Our patients with continuously increased cTNI values also had elevated lyso-Gb_3_ levels, but due to the small patient sample and since Gb_3_ values were not determined systematically in all our patients, we were not able to relate reliably cTNI levels to results of mutational analysis and lyso-Gb3 values.

Notably, all 3 FD subjects with cTNI elevations received ERT. Since these patients started ERT before first cTNI determination, it is not clear whether their cTNI values increased during or remained elevated despite ERT. However, the impact of a continuous cTNI release from cardiomyocytes under ERT deserves particular attention, since it can be speculated that ERT in its current form is not sufficient to avoid ongoing cardiac damage in such patients [33]. In addition, longitudinal cTNI determination or assessment of the so-called high sensitive troponin could serve to assess the therapeutic effect of ERT on the heart [34].

In summary, continuous cTNI release seems not to be infrequent in FD, and occurred in this study cohort exclusively in subjects with evidence of cardiac involvement. In FD, a persistent cTNI elevation appears to be primarily related to direct cardiac muscular injury by Gb_3_ deposition in myocytes. Further studies with larger numbers of patients are required to determine whether cTNI is a valid surrogate marker of cardiac involvement in FD.
